# Supplementary material for: Meanness trumps language: Lack of foreign language effect in early bilinguals’ moral choices
Source: PLoS One. 2023 Nov 30;18(11):e0294523. doi: 10.1371/journal.pone.0294523 (PMC10688712; doi:10.1371/journal.pone.0294523)
Supplement: Attachment — Submitted filename: Responses to reviewers.docx [file pone.0294523.s001.docx]

Dear Anastassia Zabrodskaja (Academic Editor), Farzaneh Shakki, Andres Molero-Chamizo, and anonymous reviewer,

We sincerely appreciate the valuable feedback you provided. We have carefully revised and enhanced our manuscript to address each point raised. Below, we provide a detailed response to each suggestion, outlining the specific changes we have made.

# **Journal requirements**

1. Please ensure that your manuscript meets PLOS ONE's style requirements, including those for file naming. The PLOS ONE style templates can be found at <https://journals.plos.org/plosone/s/file?id=wjVg/PLOSOne_formatting_sample_main_body.pdf> and [https://journals.plos.org/plosone/s/file?id=ba62/PLOSOne_formatting_sample_title_authors_affiliations.pdf](https://urldefense.com/v3/__https:/journals.plos.org/plosone/s/file?id=ba62*PLOSOne_formatting_sample_title_authors_affiliations.pdf__;Lw!!D9dNQwwGXtA!UMQqf69Y0to35ArsmGOPv7QDQG6k8L5-WafOMvINt8q3ASOAmNQsF4fny2iHp23HtfmkaLyOA064rjkkIBhL$)

**Response:** Thank you for your feedback. We have thoroughly reviewed our manuscript to ensure that it complies with the prescribed formatting for both the main body and the title/authors/affiliations sections. We apologize for any oversight regarding the file naming requirements. We renamed the figures as "Fig1.tif" and "Fig2.tif". If there are any further specific file naming guidelines you could provide or clarify, we would be more than willing to make the appropriate adjustments accordingly.

2. Thank you for stating the following financial disclosure: “This work is part of the projects EDU2017-85909-P and PID2021-123770OB-I00, founded by MCIN/AEI/10.13039/501100011033/FEDER, UE.” Please, state what role the funders took in the study.  If the funders had no role, please state: "The funders had no role in study design, data collection and analysis, decision to publish, or preparation of the manuscript." If this statement is not correct you must amend it as needed. Please include this amended Role of Funder statement in your cover letter; we will change the online submission form on your behalf.

**Response:** Thank you for the opportunity to address the lack of information on our financial disclosure. We have incorporated your suggested statement for the role of funders in the Cover Letter of this revised submission: "The funders had no role in study design, data collection and analysis, decision to publish, or preparation of the manuscript."

3. We noted in your submission details that a portion of your manuscript may have been presented or published elsewhere. [A very preliminary analysis of the study presented in the manuscript was communicated via poster at the 1st Meeting of the Society for the Advancement of Judgment and Decision Making Studies (Palma, 2016). The poster can be accessed in the following Open Science Framework (OSF) link (not anonymized): [https://osf.io/68m2y/?view_only=25d2a1cfbdcf4c349cf10ebebb815104](https://urldefense.com/v3/__https:/osf.io/68m2y/?view_only=25d2a1cfbdcf4c349cf10ebebb815104__;!!D9dNQwwGXtA!UMQqf69Y0to35ArsmGOPv7QDQG6k8L5-WafOMvINt8q3ASOAmNQsF4fny2iHp23HtfmkaLyOA064riva_CUI$) ] Please clarify whether this [conference proceeding or publication] was peer-reviewed and formally published. If this work was previously peer-reviewed and published, in the cover letter please provide the reason that this work does not constitute dual publication and should be included in the current manuscript.

**Response:** The preliminary analysis of the study presented in the manuscript was communicated via a poster at the 1st Meeting of the Society for the Advancement of Judgment and Decision Making Studies (SEJyD). However, no portion of our manuscript was published. The SEJyD presentation involved a sample of 25 participants (instead of 52) and utilized a distinct statistical procedure. It underwent a peer-review process as part of the conference proceedings, but they have not been formally published. Only the abstract was included in an abstracts booklet for the conference available at: <https://www.researchgate.net/publication/308969031_Booklet_of_the_First_SEJyD_Meeting_2016_Palma_Spain_Society_for_the_Advancement_of_Judgment_and_Decision_Making_Studies>. The current manuscript presents a comprehensive and refined version of the study, encompassing a broader sample and employing an advanced analytical approach to yield more conclusive results.

Thank you for considering this clarification.

4. We note that Figure 1 in your submission contain copyrighted images. All PLOS content is published under the Creative Commons Attribution License (CC BY 4.0), which means that the manuscript, images, and Supporting Information files will be freely available online, and any third party is permitted to access, download, copy, distribute, and use these materials in any way, even commercially, with proper attribution. For more information, see our copyright guidelines: [http://journals.plos.org/plosone/s/licenses-and-copyright](https://urldefense.com/v3/__http:/journals.plos.org/plosone/s/licenses-and-copyright__;!!D9dNQwwGXtA!UMQqf69Y0to35ArsmGOPv7QDQG6k8L5-WafOMvINt8q3ASOAmNQsF4fny2iHp23HtfmkaLyOA064rjdZ0XWu$). We require you to either (1) present written permission from the copyright holder to publish these figures specifically under the CC BY 4.0 license, or (2) remove the figures from your submission: You may seek permission from the original copyright holder of Figure 1 to publish the content specifically under the CC BY 4.0 license. We recommend that you contact the original copyright holder with the Content Permission Form ([http://journals.plos.org/plosone/s/file?id=7c09/content-permission-form.pdf](https://urldefense.com/v3/__http:/journals.plos.org/plosone/s/file?id=7c09*content-permission-form.pdf__;Lw!!D9dNQwwGXtA!UMQqf69Y0to35ArsmGOPv7QDQG6k8L5-WafOMvINt8q3ASOAmNQsF4fny2iHp23HtfmkaLyOA064rpKMrP3q$)) and the following text: “I request permission for the open-access journal PLOS ONE to publish XXX under the Creative Commons Attribution License (CCAL) CC BY 4.0 ([http://creativecommons.org/licenses/by/4.0/](https://urldefense.com/v3/__http:/creativecommons.org/licenses/by/4.0/__;!!D9dNQwwGXtA!UMQqf69Y0to35ArsmGOPv7QDQG6k8L5-WafOMvINt8q3ASOAmNQsF4fny2iHp23HtfmkaLyOA064rrbwkys-$)). Please be aware that this license allows unrestricted use and distribution, even commercially, by third parties. Please reply and provide explicit written permission to publish XXX under a CC BY license and complete the attached form.”

**Response:** Thank you for your guidance regarding the copyrighted images in our submission. We have acted upon your recommendation and removed the copyrighted figures from our manuscript. The remaining content (Fig1) includes only a comment about the 1 to 9 scale that does not involve copyrighted material. We hope that it is OK. If not, we surely will appreciate if you let us remove the Fig1, renaming Fig2 and the newly created (following Reviewer #1 suggestion) Fig3.

## **Review Comments to the Author**

### **Reviewer #1:**

- Thank you for conducting this research and for the analyses you have done. In my point of view, this manuscript is not germane to the journal scope and it can be submitted in a political journal. Your paper quality is fine, and you have met the standards of the academic writing, but it is not suitable to be published here. I do recommend you to find another journal which scope is in harmony with your purpose of study.

**Response:** Many thanks for your recommendation. We sincerely appreciate your time and effort in reviewing our manuscript. While we understand your perspective, we would like to highlight that the scope of PLOS one is intentionally broad. Moreover, we cited in our manuscript two previous papers addressing similar topics that have been successfully published in PLOS One (e.g., Costa et al., 2014; Behnke et al., 2020). Expanding the literature for the revised manuscript, we included a few more references published in PLOS One (e.g., Geipel et al., 2015; Kirova et al., 2023). Therefore, we believe that our paper fits the aims of the journal and, following all the reviewer’s suggestions, it could be published in PLOS One.

*References*

Behnke A, Strobel A, Armbruster D. (2020) When the killing has been done: Exploring associations of personality with third-party judgment and punishment of homicides in moral dilemma scenarios. *PLoS ONE*, 15: e0235253.

Costa A, Foucart A, Hayakawa S, Aparici M, Apesteguia J, Heafner J, et al. (2014) Your Morals Depend on Language. *PLoS ONE*, 9: e94842. doi:10.1371/journal.pone.0094842

Geipel, J., Hadjichristidis, C., & Surian, L. (2015). The foreign language effect on moral judgment: The role of emotions and norms. PloS One, 10(7), Article e0131529. [https://doi.org/10.1371/journal.pone.0131529](https://urldefense.com/v3/__https:/doi.org/10.1371/journal.pone.0131529__;!!D9dNQwwGXtA!UMQqf69Y0to35ArsmGOPv7QDQG6k8L5-WafOMvINt8q3ASOAmNQsF4fny2iHp23HtfmkaLyOA064roYCt_DU$)

Kirova, A., Tang, Y., & Conway, P. (2023). Are people really less moral in their foreign language? Proficiency and comprehension matter for the moral foreign language effect in Russian speakers. *PloS One, 18(7)*, Article e0287789.

- The next point is that, for your next submission, I ask you to add some graphs or charts in which you show your results to make it more readable.

**Response:** We appreciate your suggestion to enhance the readability of our manuscript. We have studied which graphs or charts could be included to visually present our results, and we think that our Fig2 illustrates the key result of our study. However, we created a new figure (Fig3) that we hope captures and illustrates other important results. Thank you for your comment.

### **Reviewer #2:**

- The Foreign-Language (FLe) effect on moral judgments in early bilinguals is evaluated in this study, as well as the potential role of personality traits and emotional load in this type of judgments. The authors conclude that FLe is not evident in early bilinguals, and cruelty is a personality trait than can modulate moral judgements and decisions, regardless of language or dilemma type. They also found significant interactions between some characteristics of dilemmas and personality traits. The manuscript is well written, the design and methodology are appropriate, and the overall discussion is well grounded in the results. The results are interesting for publication. However, there is something that should be discussed in the article before publication.

**Response:** Many thanks for your positive comments that we really appreciate.

- In my opinion the two languages evaluated in this study are very similar (Spanish and Catalan), and they share identical or similar words. In contrast, FLe studies analyze very different languages (English vs. Dutch, or English vs. Chinese, for example). This limitation affecting the results should be critically discussed in the manuscript, and the conclusions from the results should be tone down accordingly. I add here some comments that also might improve the quality of this manuscript.

**Response:** Many thanks for the suggestion. In fact, testing the FLe in bilinguals having similar languages learned both at a very early age is one of the aims of the present work, and we consider this a strength of our works due to its novelty. While prior FLe studies have predominantly focused on analyzing significantly different languages, our investigation sheds light on the impact of subtle linguistic variations between closely related languages. Indeed, we have now added new paragraphs in which we discuss the linguistic dynamics and the similarities between Catalan and Spanish (e.g., page 6 lines 137-158, page 26 lines 565-578, of the clean copy of the revised manuscript).

For instance, Catalan and Spanish are both Romance and transparent languages sharing a large number of cognate words with similar phonetics and semantics, which has been estimated to be 76% (Lewis et al., 2014). Furthermore, exposure to Catalan and Spanish in the region where the sample was recruited is considerably balanced in terms of usage, being present at educational, social, and media settings. Children often learn both languages simultaneously when they are very young. Moreover, the social environment usually facilitates the dynamic use of both languages in these Catalan-speaking provinces,

As it is very common in the Balearic Islands, participants considered that Catalan was their mother tongue with a high proficiency, while also having a very high proficiency in Spanish, their L2. In fact, Spanish is a language effortlessly acquired across different sub-groups of speakers immersed in our stable bilingual community (Gathercole & Thomas, 2009).

*References*

Lewis, MP, Simons, GF and Fennig, CD (2014) *Ethnologue*, 17th ed. Dallas: SIL International.

Gathercole, VCM and Thomas, EM (2009) Bilingual first-language development: Dominant language takeover, threatened minority language take-up. *Bilingualism: Language and Cognition* 12, 213–237. <https://doi.org/10.1017/>S1366728909004015

- Abstract: The authors may considerer the possibility to clarify from the abstract if moral dilemmas were displayed on paper or by computer, and it would also be interesting to know here if the response was dichotomous (yes or no) or otherwise.

**Response:** Thank you for your constructive suggestion. We have taken your feedback into consideration and clarify in the abstract that the moral dilemmas were displayed on a computer. Additionally, we have specified that the participants' responses were dichotomous in nature (utilitarian vs deontological).

*Introduction*

- In the Introduction section, the authors should explain the reason to include two similar languages when evaluating the FLe. Have been previously compared these two close related languages in other FLe studies? In my opinion this limitation is critical for understanding the results.

**Response:** Thank you for your valuable feedback. In response to your comment, we have incorporated a paragraph in the introduction (page 6 lines 137-158 of the clean copy of the revised manuscript) that outlines the rationale for examining closely related languages, specifically Spanish and Catalan. We acknowledge the shared linguistic characteristics, including vocabulary and structural similarities, between these languages. Our investigation of language pairs with subtle variations aims to contribute a unique dimension to the understanding of language effects on cognitive processes. Also, it is important to note that several studies cited in our paper focused on assessing the FLe in nearby languages like Dutch and English (Brouwer, 2019) and Swedish and Norwegian (Dylman et al., 2020), which are German languages. Our research extends this inquiry to the unique linguistic dynamics present between two closely related Romance languages in a dual-language context. Now we highlight this also in the introduction (page 6 lines 144-158 of the clean revised manuscript). Furthermore, we have extended a little bit the discussion section to acknowledge this specific context (page 27 lines 571-578 of the clean revised manuscript).

*References*

Brouwer, S. (2019). The auditory foreign-language effect of moral decision making in highly proficient bilinguals. *Journal of Multilingual and Multicultural Development*; 1–14. https://doi.org/10.1080/01434632.2019.1585863

Dylman, AS, Champoux-Larsson, M-F. (2020). It’s (not) all Greek to me: Boundaries of the foreign language effect. *Cognition* 196: 104148.

- Page 3, lines 53-55: I am not sure if this is the best description of the nature and content of moral dilemmas used in research: “This kind of stimuli describe a situation in which two moral reasons are contraposed: for instance, not to kill (deontological outcome) versus maximizing welfare by any means (utilitarian outcome).” To understand what exactly means a moral dilemma in moral judgments studies, the authors should improve the provided description of moral dilemmas task, or the meaning and utility of this research tool will be lost.

**Response:** To be honest, with every draft, we refined the introduction to maintain it as focused, brief, and concise as possible, explaining only what was essential to our argumentation. However, we recognize the importance of providing a clear and precise depiction of the nature and content of the stimuli. Thus, we revised our description (page 3 lines 56-60 of the clean revised manuscript) defining moral dilemmas as situations "in which participants are confronted with conflicting moral reasons, such as the contrast between adhering to a deontological principle (e.g., not to kill) versus maximizing overall welfare through utilitarian means (e.g., kill one to save many)." We think that our cited references provide yet in-depth understanding of moral dilemmas (e.g., Christensen et al., 2014). Therefore, we believe that now the introduction is more accurate, maintaining the balance between brevity and guidance for those who wish to delve deeper into the concept of moral dilemmas. Thank you for pointing it out to us.

*References*

Christensen JF, Flexas A, Calabrese M, Gut NK, Gomila A. (2014) Moral judgment reloaded: a moral dilemma validation study. *Front Psychol*. 1;5:607. doi: 10.3389/fpsyg.2014.00607.

- Page 3, lines 61-63: The authors should clarify that personal dilemmas are usually considered high conflict dilemmas in the moral judgement studies, and impersonal dilemmas are considered low conflict dilemmas. This clarification is in line with the terminology commonly used in this area. This is important when reporting in the results section that impersonal dilemmas (that is, low conflict dilemmas) are more associated with utilitarian responses.

**Response:** We have taken your suggestion into consideration, and we have revised the introduction to clarify the distinction between personal dilemmas, typically considered high conflict dilemmas in moral judgment studies, and impersonal dilemmas, often categorized as low conflict dilemmas (page 3 lines 66-72 of the clean revised manuscript). However, we think that this terminology usage within the broader literature on FLe and moral dilemmas is not uniformly established and, given the specific nature of our study, we acknowledged that a nuanced discussion of terminology, while important, may not be the primary focus of our manuscript. We believe that the incorporation of this clarification enhances the reader's understanding of the terms used in our study, while ensuring that the central themes of FLe and psychopathy remain at the forefront of our exploration. Thank you again for your valuable input.

- Page 4, lines 83-85: The authors state: “Nevertheless, it seems plausible to say that, for early and proficient bilinguals, L2 should be as emotional as L1; thus, FLe should be absent, because L2 is not a foreign language for simultaneous early bilinguals”. This hypothesis makes sense, but I insist that in this study there is another factor to be considered. L2 might not be a foreign language to the participants of this study as they are early bilinguals, as the authors state, but, most importantly, both languages share words, origins, and phonological and syntactic structures that make them languages not clearly differentiated. This last factor is something that clearly differs from the FLe studies, and should be treated accordingly in this study. In lines 86-87 the authors superficially mention this possibility (“linguistic similarity”), but it should be explicit from the introduction (and detailed in the discussion section).

**Response:** Thank you for your comment. As we previously responded, we acknowledge the importance of considering the linguistic similarities between the languages under investigation. Indeed, we have revised our introduction and discussion sections (as indicated above), highlighting the unique nature of our study, which examines the absence of the FLe within the context of languages that share linguistic characteristics to a greater extent than previous literature. However, we think that the lines you mentioned must remain as they are in order to maintain the introduction focused. Thank you again for emphasizing the significance of this factor and for recognizing our efforts in addressing this concern. Your input has played a crucial role in enhancing the depth and accuracy of our manuscript's presentation and analysis.

- Page 5, lines 109-110: “when the person who is killed were going to die anyway”; there is a typo here (the person… were…).

**Response:** Thank you for pointing out the typo in our manuscript. We appreciate your attention to detail. We have corrected the error to read: "when the person who is killed was going to die anyway" (page 6 line 128 of the clean revised manuscript)

- Page 5, line 118: “…are thus of particular interest”. Is “are” referring to additional research? In this case, should not be “is thus of…”?

**Response:** You are correct, and we apologize for the grammatical oversight. The correct phrasing should be: "...is thus of particular interest" (page 6 line 136 of the clean revised manuscript). We appreciate your attention to detail.

- Page 6, lines 141-142: “…thus, personal dilemmas will yield quicker responses than impersonal ones”. I would say that it depends; personal dilemmas being high conflict dilemmas should not be related with faster responses. Maybe the authors mean that personal dilemmas with low conflict will have faster responses. In fact, DT (Reaction Time for decision) should be analyzed differentially in low and high conflict dilemmas, and in personal and impersonal dilemmas. This should be the correct approach to elucidate if DT is affected according to the different dilemma combinations, that is, personal vs. impersonal and high vs. low dilemmas. In case that the authors only use in their study personal dilemmas with low conflict (which on the other hand is not usual), this should be clarify. In most studies, personal dilemmas involve high conflict.

**Response:** Thank you for your detailed explanation and insights. Your interpretation of our statement is accurate, and we appreciate your clarification regarding the potential confusion around personal dilemmas and their varying degrees of conflict. However, we would like to emphasize that the variation in conflict levels within personal dilemmas depends on other factors, including benefit recipient (self vs others), avoidability of death (avoidable vs unavoidable), and intentionality (accidental harm vs instrumental harm). In our study**,** as stated in the Methods section, we aimed to create a balanced selection of moral dilemmas within specific categories such as personal force, benefit recipient, and avoidability of harm (all the scenarios depicted instrumental harm, so intentionality was blocked). Given the constraints of scenario quantity, we acknowledge that our approach has limitations that may impact the depth of analysis in certain aspects, and we greatly appreciate your understanding of this aspect. Taking that into account, our approach may allow us to maintain the hypothesis that personal dilemmas yield quicker responses, while recognizing the complexity introduced by factors like benefit recipient and avoidability. We have now highlighted this in the discussion section (page 29 lines 622-634 of the clean revised manuscript). Thank you again because your careful consideration of our methodology is instrumental in refining the accuracy and depth of our research.

- Page 6, lines 143-145: “…we expect no differences between responses to moral dilemmas presented in L2 with respect to L1, as our participants are high proficient early bilinguals…” Again, not only for that reason, but also because the languages evaluated in this study are closely related phonological and syntactically, unlike typical FLe studies (English vs. German, Chinese, etc.). Spanish is considered in this study L2, but participants are actually all Spanish, and it sounds that here there is not a real distinction between L1 and L2. Actually, all of them learned both languages before 3 years of age. How can be distinguished L1 and L2 under this language learning circumstances?

**Response:** We have taken your feedback into careful consideration and have revised the paragraph acknowledging that these languages are closely related, but not the same language (page 8 lines 185-190 of the clean revised manuscript). It is true that Catalan and Spanish share phonological and syntactic characteristics and are both transparent languages that have been acquired at an early age by our participants, making the traditional distinction between L1 and L2 less clear or evident. Despite this complexity, we continue to refer to Spanish as the L2 in our study due to the bilingual context of our participants and the facts that: (1) participants considered Catalan as their L1 and (2) self-reported proficiency in L1 was higher than in L2.

This bilingual context is not necessarily determinant because there could be people that, although consider themselves bilinguals, they did not consider themselves equally proficient in both languages. In fact, all of our participants considered that they have a native language (L1 - Catalan) and an early acquired yet distinct (not native) language (L2 - Spanish). Moreover, now we report the *t*-test for proficiency scores (page 10 lines 237-238 of the clean revised manuscript) that confirms that there are significant differences between proficiency in both languages. Even while our participants are highly proficient in both languages and acquired them at an early age, data supports that they are dealing with two distinct languages. Therefore, now we highlight in the text that, while early bilinguals can attain high proficiency in both languages, their relative proficiency in L1 and L2 can be influenced by contextual factors (page 11 lines 238-240 of the clean revised manuscript).

- *Participants*
  I am not sure if participants with age of 46 years face the task of this study as students between 18-23 years old would do. Are there statistical comparisons between age sub-groups?

**Response:** Thank you for your feedback. It is indeed a valid consideration to ponder whether individuals from different age groups might approach tasks differently. However, our study did not involve specific statistical comparisons between age sub-groups. As you correctly noted, our sample predominantly consists of participants between the ages of 18 and 23, with only one participant aged 46 years. We think that, although age can be a potential variable of interest, exploring potential age-related differences is not our aim, although could provide valuable insights in future research.

- Participants were considered themselves bilingual… Was this confirmed by the authors, apart from the self-reported scales?

**Response:** Participants' self-reported bilingualism was indeed a primary criterion for inclusion in the study Although language level was not objectively confirmed using other measures than a self-report, we are very confident that all participants were high proficient bilinguals due to several reasons. First, they all reported to have a high proficiency in the oral and written domains, both in the productive and the receptive areas. Second, all participants were university students. This situation obliges all participants to have a high degree of knowledge in both languages, given that the entrance exam to the University includes language level tests in both languages. Also, university classes are taught in both Catalan and Spanish, and even the same subject has professors who teach their parts either in Catalan and/or Spanish, as Catalan and Spanish are both co-official languages. Finally, using self-reports without objective measures of language level –although we concur that this is a caveat and might have its limitations– is a common practice in studies addressing the FLe (see Brouwer, 2019; Costa et al., 2014a; Costa et al 2014b; Dylman & Champoux-Larsson, 2020; Keysar, Hayakawa & An, 2012; Woumans, Van der Cruyssen & Duyck, 2020, among others).

*References*

Brouwer, S. (2019). The auditory foreign-language effect of moral decision making in highly proficient bilinguals. *Journal of Multilingual and Multicultural Development*, *40*(10), 865-878. doi:10.1080/01434632.2019.1585863

Costa, A., Foucart, A., Hayakawa, S., Aparici, M., Apesteguia, J., Heafner, J. et al. (2014). Your Morals Depend on Language. *PLoS ONE*. *9,* e94842. doi:10.1371/journal.pone.0094842

Costa, A., Foucart, A., Arnon, I. Aparici, M., Apesteguia. (2014). “Piensa” twice: On the foreign language effect in decision making. *Cognition*. *130*(2)*, 236-254*. doi:10.1016/j.cognition.2013.11.010

Dylman, A., & Champoux-Larsson, M. F. (2020). It’s (not) all Greek to me: Boundaries of the foreign language effect. *Cognition*, *196*. doi:10.1016/j.cognition.2019.104148

Keysar, B., Hayakawa, S. L., & An, S. G. (2012). The Foreign-Language Effect: Thinking in a Foreign Tongue Reduces Decision Biases. *Psychological Science*, *23*(6), 661–668. <https://doi.org/10.1177/0956797611432178>

Woumans, E., Van der Cruyssen, I., & Duyck, W. (2020). Crime and punishment: Morality judgment in a foreign language. *Journal of experimental psychology. General*, *149*(8), 1597–1602. doi:10.1037/xge0000736

- Page 8, lines 190-191: “Participants were proficient in both languages, M = 3.67, SD = 0.37, and M = 3.25, SD = 0.57 for L1 and L2 overall proficiency, respectively”. This slightly suggests which one could be L1, but these results are based on self-reported scales. This limitation should be seriously discussed.

**Response:** We concur that the use of self-reports as the unique source of information can be considered as a limitation of the study, and we have now made this explicit in the manuscript (page 32 lines 709-715 of the clean revised manuscript). Although we fully agree with the Reviewer’s point, we would like to note that all participants considered themselves bilinguals having Catalan as their native language; additionally, self-reported proficiency was slightly –but significantly– larger in L1 than in L2.

- Page 9, line 200: “…in accordance with the Declaration of Helsinki”. The last one?

**Response:** Certainly, we ensured that our study was conducted in accordance with the principles outlined in the most recent Declaration of Helsinki (2013), as it is the currently valid version.

- Page 9, lines 201-202: “Authors had no access to information that could identify individual participants during or after data collection”. Therefore, the participation in this study was anonymous, and thus should be reported.

**Response:** Thank you for your comment. We have now explicitly included in the abstract that participation in this study was anonymous (page 2 line 28 of the clean revised manuscript).

- *Materials*
  Page 10 (moral dilemmas task), lines 219-220: “In each of these two categories, four of the eight scenarios were self-beneficial (SB) and four were other-beneficial dilemmas (OB)”. Does this mean low and high conflict dilemmas? What were low and high conflict dilemmas should be clarify.

**Response:** Thank you for raising this concern. We apologize for any confusion caused. To clarify, the self-beneficial (SB) and other-beneficial (OB) categorization pertains to the recipient of the benefit within the moral dilemma scenarios. As you correctly noted, we could expect lower internal conflict in SB scenarios but, as we commented above, internal conflict did not emerge from this factor (Benefit Recipient) but its interaction with Personal Force and other factors like Avoidability and Intentionality. We think that our edited paragraph in the Discussion (page 29 lines 622-634 of the clean revised manuscript) is in line with this rationale: "This indicates that benefit recipient and avoidability jointly have a similar effect on moral choice than personal force. Interestingly, there were no interactions with personal force, despite the expectation that these factors might contribute to variations in the degree of conflict within personal dilemmas, which are traditionally considered to involve high conflict. While our approach may have limitations in representing the full spectrum of high and low-conflict dilemmas, future studies on moral decisions should not underestimate the effects of benefit recipient, and avoidability of death, taking these variables into account when studying the impact of personal variables, like psychopathic traits [41,67]. It is also noteworthy to remember that our design blocked intentionality in instrumental harm (it could be the reason why arousal and valence are very similar between the personal and impersonal versions of dilemmas). Thus, intentionality could be another key factor in varying the degree of conflict of the dilemmas and future studies should address this variable as well”.

- Page 10, lines 228-229: I was not able to access to the files by the link [https://osf.io/4vry5/?view_only=597aa6dab16c43dd9d32b6036691958b](https://urldefense.com/v3/__https:/osf.io/4vry5/?view_only=597aa6dab16c43dd9d32b6036691958b__;!!D9dNQwwGXtA!UMQqf69Y0to35ArsmGOPv7QDQG6k8L5-WafOMvINt8q3ASOAmNQsF4fny2iHp23HtfmkaLyOA064rvendVVB$)
  Are they really available?

**Response:** Thank you for bringing this to our attention. We apologize for any inconvenience you experienced while trying to access the files through the provided link. The files are indeed available on the OSF, although perhaps we failed to make it publicly available. If the issue persists, please let us know, and we'll be happy to assist you further in accessing the files. We appreciate your understanding and patience in this matter. Anyway, we have created a new link, not anonymous, hoping that it will solve any problem: <https://osf.io/4vry5/?view_only=97832849863f465d9fe79d925a63b306>

- *Procedure*
  Page 11, line 236: This is the first time that readers can know if dilemmas were displayed on paper or otherwise. In my opinion, this information should be briefly mentioned from the beginning (the abstract), as previous studies have generally used a dilemma presentation in paper format.

**Response:** As stated above, we revised the abstract. Now the readers are informed about the presentation format of the moral dilemmas from the outset (page 2 line 28 of the clean revised manuscript). Thank you for helping us to refine the accessibility and clarity of our study's presentation.

- Page 11, lines 239-241: “A block of 10 dilemmas in Catalan and a block of 10 dilemmas in Spanish, counterbalancing the order of language blocks across participants, were presented by E-Prime 2.0 [44] to all participants”. Do you mean counterbalancing the order of language blocks and in a randomized way across participants? Otherwise, each counterbalancing result (Spanish-Catalan, and Catalan-Spanish) is supposed to be presented continuously in half of participants (first S-C and then C-S, or the other way).

**Response:** Thank you for bringing up this important aspect of our study's methodology. We appreciate the opportunity to clarify the counterbalancing process that we applied.

In order to mitigate potential order effects, we implemented a counterbalancing strategy that ensured a randomized presentation of language blocks and dilemma versions across participants. Each participant was assigned to one of the four possible sequences (e.g., Catalan-Personal/Spanish-Impersonal, Catalan-Impersonal/Spanish-Personal), and each sequence was then assigned to approximately an equal number of participants. This approach aimed to minimize any potential bias introduced by the order of language blocks and dilemma versions. We hope that the new OSF link provided (<https://osf.io/4vry5/?view_only=97832849863f465d9fe79d925a63b306>) will be useful in order to understand this procedure, as we originally included a document ("Dilemma task additional info") addressing this issue. If you find that it is insufficient, we are open to enhancing the clarity of this aspect within the Methodology section.

- Page 11, lines 249-250: “responding by pressing I key (meaning "yes") or O key (meaning "no")”. Can the authors explain why these keys were used? Responses were made with a particular hand (right or left) or the participants responded indistinctly with either hand. This is important, among other things, for RT (DT).

**Response:** Thank you for your query. We decided to use these keys because the letters “i” and “o” are part of the Catalan (and Spanish) “Sí” and “No” words (“Yes” and “No”), so the keys are intuitively related to the response they represent. Moreover, these keys are located side by side in a Spanish QWERTY keyboard, allowing participants to respond comfortably with one hand. Precisely, to address potential implications of hand preferences for DT, we asked about participant’s hand preference and they responded with their dominant hand. It is now explained in the reviewed manuscript (page 14, line 308-315).

- Page 12, line 261: again, not files can be downloaded from the link provided by the authors:  <https://osf.io/4vry5/?view_only=597aa6dab16c43dd9d32b6036691958b>

**Response:** Thank you for bringing this to our attention. We apologize again for any difficulties you have encountered while trying to access the files through the provided link. As said before, you can now access the supplementary materials for our study at the following link: <https://osf.io/4vry5/?view_only=97832849863f465d9fe79d925a63b306>. If you continue to face any issues, please let us know.

- *Statistical analyses*
  Page 12, lines 277-278: “Responses to moral dilemmas with DT < 1000 ms (2.4% of trials) were excluded from the analyses”. That means that DTs of, for example, 1.1 sc were included. Is this time enough to read the question (two lines according to Fig. 1) and take the decision?

**Response:** We sincerely appreciate your thoughtful consideration of the reaction time (RT) threshold implemented in our study. The decision to exclude responses with DT < 1000 ms was driven by the primary objective of mitigating inadvertent anticipations, such as unintentional key presses, while upholding the data's integrity.

It is worth highlighting that all essential information found in the question screen has already been comprehensively presented in the preceding dilemma screen. Toward the conclusion of the dilemma screen, the fundamental premises underlying the decision at hand have been distinctly articulated. Consequently, the question screen introduces no novel information; instead, it poses a succinct query naturally derived from the context thoughtfully established in the preceding phase, rendering it readily comprehensible.

Example:

Last paragraph of the “shark” dilemma (dilemma screen):

“If you shoot at the injured diver this will kill him and the sharks will stop to eat him, but you and the ten divers will be saved.”

Question of “shark” dilemma (question screen):

“Do you let the sharks eat the injured diver by shooting at him, so you and the other ten divers can reach the protective cage?”

- Page 16, Table 3 legend: “a: One participant answered that he/she was slightly more proficient in L2. It seems a mistake but some early bilinguals in Mallorca consider that they are equally or more proficient in L2 because their true mother tongue differed from the Catalan normative language”. Therefore, it is not clear even to the participants which one is L1 and L2, and the interpretation of the results of this study should consider this limitation.

**Response:** Thank you for highlighting again this important aspect. We appreciate your understanding and acknowledgment of the complexities surrounding the interpretation of our results. Indeed, as we indicated above, the distinction between L1 and L2 in the context of Mallorca depend on the language used at home with the family (native language - L1) but L1 it is not necessarily the language in which the participant consider her/himself most proficient, although this was the case for all participants except one in the present study. In fact, numerical data for this participant shows that he/she felt more proficient in his/her L2 because he/she felt more comfortable in written expressive proficiency in Spanish, thus making the total proficient score higher in L2. This might be influenced by the broad use of and exposure to Spanish in media settings in our dual language context.

We agree that this is a limitation of our study and, precisely, we think that the note in the Table 3 (now revised for clarity ­–page 18 lines 361-362) make readers aware of the potential ambiguity in participants' self-perceived language proficiency. We hope this clarification and the improved paragraphs in the Discussion following your comments will help in understanding and contextualizing our findings.

- *Discussion*
  Please, consider the limitation regarding the similarity of both languages when discussing the results related to the FLe.

**Response:** Certainly, we appreciate your reminder to consider this limitation. As stated above, we have revised our Discussion section to explicitly address this limitation and its potential implications for the interpretation of our findings in the context of the FLe, thus toning down the extent of our conclusions. Thank you again for highlighting this aspect.

- Page 28, lines 599-601: “Future studies could address these limitations by using larger and more diverse samples or investigating other factors that could affect moral decision-making, such as cultural background or education level”. The authors may want to discuss the cultural and educational effect on moral decisions in line with the results reported in the following study:
  [https://www.tandfonline.com/doi/full/10.1080/00049530.2021.1882276](https://urldefense.com/v3/__https:/www.tandfonline.com/doi/full/10.1080/00049530.2021.1882276__;!!D9dNQwwGXtA!UMQqf69Y0to35ArsmGOPv7QDQG6k8L5-WafOMvINt8q3ASOAmNQsF4fny2iHp23HtfmkaLyOA064rlzdYqpI$)
  Moreover, a gradation in the responses (which were not dichotomous) can be found in that study, which has not been previously evaluated in the moral dilemma literature. The authors may also want to discuss this procedural difference (a graduation in the responses) in regards to their results.

**Response:** We appreciate your suggestion and the reference to the study highlighting cultural and educational effects on moral decisions. While we did not specifically investigate cultural and educational factors in our study, the link you provided offers valuable insights into this area (we have now included it in the Discussion –page 32 lines 708-715). Regarding the procedural difference you mentioned, we opted for dichotomous responses for several reasons. Rather than assessing the level of moral appropriateness or gradation in the decision, our question inquires whether the action is taken or not (it is not asked in the conditional, we asked "Do you do it?"). It requires a "Yes"/ "No" response. We think that this dichotomous approach aligns with the essential nature of a moral dilemma involving a distinct choice. It's also worth noting that this procedure is commonly used in the literature focusing on the FLe and psychopathy. Nonetheless, we acknowledge that the use of a gradation in responses could provide an interesting avenue for future exploration, potentially offering additional nuances in understanding participants' decision-making processes. Thank you for highlighting this procedural consideration and its potential implications for future research.

- A last minor thing. The short title is “MEANNESS AND UTILITARIAN JUDGMENTS IN EARLY BILINGUALS”, but I think that utilitarian are the responses (or solutions to dilemmas), not the judgements themselves.

**Response:** Thank you for your careful reading and observation. We appreciate your attention to detail. We agree with your point that the term "utilitarian" more accurately refers to the responses or choices to the moral dilemmas rather than the judgments themselves. To better reflect the content of our study, we will revise the short title to "MEANNESS AND MORAL CHOICES IN EARLY BILINGUALS." This adjustment will align the title with the focus of our investigation. We are grateful for your input, which has contributed to enhancing the precision and clarity of our manuscript.

### **Reviewer #3:**

- This study investigates a relevant topic (decision-making and individual differences). However, it tries to combine so many variables that it is difficult to maintain the focus and overall coherence and to provide an in-depth interpretation of the results. The novelty of the study is not clear; it appears that replicating previous findings is the main aim of the study. For example, we already know that proficiency level influences decision-making. In addition, we know that for many L1 speakers of Catalan, Spanish turns out to be the language that they mostly use or are exposed to much more often than their L1, which limits the conclusions that can be derived from this study. In what follows, I provide more detailed comments.

**Response:** Thank you for your thoughtful feedback. We appreciate your recognition of the relevance of the topic and your willingness to provide detailed comments. We acknowledge your concerns about the complexity of the variables investigated and the need to maintain focus and coherence in the study. Your insights will be instrumental in guiding these improvements.

- *Introduction*
  In the introduction, the authors argue that “language can make a difference in our moral decisions … because language is unavoidably related with emotion”. That´s correct. But it is surprising the fact that the only study that the authors cite is Brouwer (2021). I believe that other studies, especially those that used mediation analyses to examine the mediating role of emotions in the link between language and moral decision-making should be acknowledged; see, for example,
  • Geipel, J., Hadjichristidis, C., & Surian, L. (2015a). How foreign language shapes moral judgment. Journal of Experimental Social Psychology, 59, 8–17. [https://doi.org/10.1016/j.jesp.2015.02.001](https://urldefense.com/v3/__https:/doi.org/10.1016/j.jesp.2015.02.001__;!!D9dNQwwGXtA!UMQqf69Y0to35ArsmGOPv7QDQG6k8L5-WafOMvINt8q3ASOAmNQsF4fny2iHp23HtfmkaLyOA064rh9BRAaI$)
  • Geipel, J., Hadjichristidis, C., & Surian, L. (2015b). The foreign language effect on moral judgment: The role of emotions and norms. PloS One, 10(7), Article e0131529. [https://doi.org/10.1371/journal.pone.0131529](https://urldefense.com/v3/__https:/doi.org/10.1371/journal.pone.0131529__;!!D9dNQwwGXtA!UMQqf69Y0to35ArsmGOPv7QDQG6k8L5-WafOMvINt8q3ASOAmNQsF4fny2iHp23HtfmkaLyOA064roYCt_DU$)
  • Kyriakou, A., Foucart, A., & Mavrou, I. (2022). Moral judgments in a foreign language: Expressing emotions and justifying decisions. International Journal of Bilingualism. [https://doi.org/10.1177/13670069221134193](https://urldefense.com/v3/__https:/doi.org/10.1177/13670069221134193__;!!D9dNQwwGXtA!UMQqf69Y0to35ArsmGOPv7QDQG6k8L5-WafOMvINt8q3ASOAmNQsF4fny2iHp23HtfmkaLyOA064ru6uOm3H$)

**Response:** Thank you for pointing out these valuable references. We apologize for the oversight in not including them in the introduction. In our revised manuscript, we incorporated these references (e.g., page 3, lines 48-53, page 6 lines 137-158, page 7 lines 159-176 of the clean copy of the revised manuscript) to provide a more comprehensive and accurate reflection for our theoretical framework. Your contribution in this regard is highly appreciated.

- The same applies to “In this vein, FLe studies with moral dilemmas [3,10] have suggested that second language provides to bilingual people a greater emotional distance than their first, native language (L1), leading to more utilitarian decisions using L2 than using L1”. Please consider adding more relevant studies.

**Response:** Thank you for your suggestion. We have expanded the list of references in the revised manuscript to include additional relevant studies, while also striving to maintain conciseness and focus.

- “It has been said that bilinguals are more emotional before a moral dilemma when…” – Why before and not after?

**Response:** Thank you for raising this point of clarification. In the context of the sentence, we used "before" to indicate the state of bilinguals when they are faced with a moral dilemma, i.e., when they are confronted with the dilemma. We appreciate your attention to this aspect of the sentence and revised the manuscript in line with this, and other of your comments, in order to be more correct and less ambiguous.

- “There is certain controversy about their presence [of the FLe] in early, high proficient, bilinguals” – That´s correct. The authors may want to include more studies that investigated the role of proficiency level, such as:
  • Corey, J. D., Hayakawa, S., Foucart, A., Aparici, M., Botella, J., Costa, A., & Keysar, B. (2017). Our moral choices are foreign to us. Journal of Experimental Psychology: Learning, Memory, and Cognition, 43(7), 1109–1128. [https://doi.org/10.1037/xlm0000356](https://urldefense.com/v3/__https:/doi.org/10.1037/xlm0000356__;!!D9dNQwwGXtA!UMQqf69Y0to35ArsmGOPv7QDQG6k8L5-WafOMvINt8q3ASOAmNQsF4fny2iHp23HtfmkaLyOA064rmh-2pmi$)
  • Kirova, A., Tang, Y., & Conway, P. (2023). Are people really less moral in their foreign language? Proficiency and comprehension matter for the moral foreign language effect in Russian speakers. PloS One, 18(7), Article e0287789.
  Based on this research, the authors must justify the contribution and novelty of their study in a more explicit and straightforward way.

**Response:** Thank you for providing these relevant references and your constructive feedback. We have expanded our references to include the studies you mentioned. We also understand the importance of clearly justifying the contribution and novelty of our study. In our revised manuscript, we emphasize how it adds to the existing literature (page 6 lines 137-176 of the clean copy). We hope these adjustments address your concerns and reflect the contribution and novelty of our research more explicitly and straightforwardly.

- *Goals of the study*
  The first goal of the study is to replicate previous findings. Please explain why this replication is important and necessary and how this replication adds to what we already know about the topic.

**Response:** Thank you for raising this question. We value replication as a crucial aspect of scientific research that contributes to the robustness and reliability of findings. It's also important to note that, in our replication, the specific context of our participants can provide valuable insights that enhance our understanding of the Foreign Language Effect (FLe) and moral decision-making. For instance:

1. Methodological Soundness and Theoretical Grounding: By replicating the tendency to respond utilitarianly to impersonal dilemmas and deontologically to personal ones, we gained confidence in our conceptual framework and our own results.

2. Cross-Linguistic Replication: Our replication of previous findings in the context of two closely related Romance languages (Spanish and Catalan), contributes to the generalizability of the FLe phenomenon. Previous literature has shown that closely related languages often share significant linguistic and cultural similarities, which can mitigate the cognitive differences observed in FLe (see the meta-analysis by Circi et al., 2021) due to either a strong cultural influence of the L1 on L2 or due to the linguistic similarities. In fact, the study of the FLe using close languages had been conducted mainly with Germanic languages, such as English, Dutch, German, Swedish, and Norwegian (see Dylman & Champoux-Larsson, 2020). However, the study by Miozzo et al. (2020) observed the FLe in close Romance languages (Italian-Venetian, Italian-Bergamasque), in early, high proficient bilinguals, and this finding contended against the mitigation of the FLe due to the linguistic similarity. The authors argued that these languages (Venetian and Bergamasque) were spoken in different contexts, namely households and informal circles, unlike Italian, thus being the context in which languages are spoken the crucial factor for the appearance of the FLe mainly when this context is different for each language.

Our aim was thus to further clarify whether the FLe was also absent in close Romance languages in early bilinguals immersed in a dual-language context (in contrast to single-language contexts, as noted by Green & Abutalebi, 2013) in which both languages are used equally in the same contexts and not being one of them restricted to informal circles. We believe this clarification will add robustness to the findings related to the FLe and will help to delimit the circumstances under which the FLe is observed, and examine other dispositional and subjective variables that could be crucial for decision-making processes.

3. Early High Proficiency Bilinguals: Our study targets early high proficiency bilinguals, a specific population that has received relatively less attention in previous research. Previous studies have shed different results showing that no language effects are to be found with late proficient sequential bilinguals (Čavar & Tytus, 2018), but see (Białek & Fugelsang, 2019). Our approach will enable us to investigate the presence of the FLe in a unique early bilingual group with distinct characteristics compared to other bilingual populations.

4. Incorporating Dilemma-Specific Factors and Psychopathic Traits: Our study goes beyond mere replication by incorporating additional dilemma-specific factors beyond personal force, such as benefit recipient, avoidability, and participant assessments. These factors can influence moral decision-making. Additionally, we explore the role of psychopathic traits (from its triarchic conceptualization), a novel variable that hasn't been extensively examined in relation to the FLe. This broadens the scope of previous research by investigating individual differences that may contribute to variations in moral judgment behaviour across languages, allowing us to delve deeper into the underlying mechanisms that drive the FLe.

In summary, while our study aims to replicate certain aspects of previous findings, the specific context, participants' group, inclusion of dilemma-specific factors, and exploration of psychopathic traits introduce novel dimensions to the research. We think that the revised introduction highlight these aspects (e.g., page 6 lines 137-176 of the clean revised manuscript).

*References*

Białek, M., & Fugelsang, J. (2019): No evidence for decreased foreign language effect in highly proficient and acculturated bilinguals: A commentary on Čavar and Tytus (2018). Journal of Multilingual and Multicultural Development, 40(8), 679–686. https://doi.org/10.1080/01434632.2018.1547072

Čavar, F. & Tytus, AE. (2018) Moral judgement and foreign language effect: when the foreign language becomes the second language, Journal of Multilingual and Multicultural Development, 39:1, 17-28, DOI: 10.1080/01434632.2017.1304397

Circi, R., Gatti, D., Russo, V. et al. (2021). The foreign language effect on decision-making: A meta-analysis. Psychon Bull Rev 28, 1131–1141. https://doi.org/10.3758/s13423-020-01871-z

Dylman, A., & Champoux-Larsson, M. F. (2020). It’s (not) all Greek to me: Boundaries of the foreign language effect. Cognition, 196. https://doi.org/10.1016/j.cognition.2019.104148

Green DW, & Abutalebi J. (2013). Language control in bilinguals: The adaptive control hypothesis. J Cogn Psychol (Hove). 2013 Aug;25(5):515-530. doi: 10.1080/20445911.2013.796377. PMID: 25077013; PMCID: PMC4095950.

Miozzo, M., Navarrete, E., Ongis, M., Mello, E., Girotto, V., & Peressotti, F. (2020). Foreign language effect in decision-making: How foreign is it?. Cognition, 199. https://doi.org/10.1016/j.cognition.2020.104245

- The second goal is to “explore how psychopathic traits and variables of the dilemmas would separately and/or jointly affect moral judgment behavior”. First, it is not clear how this goal is linked to the first one. Second, you could name the “variables of the dilemmas”. Third, please explain better what you mean by “separately and/or jointly”. Fourth, personal force, benefit recipient and avoidability have not been defined until line 130. Fifth, difficulty, verisimilitude and vividness are introduced in lines 131–132 without previous explanations of why these variables have been chosen or what the theoretical framework behind this choice is. Could you please link better this objective to your theoretical framework? Does objective 2 also aims to replicate previous findings?

**Response:** We appreciate your effort to provide clarity and coherence to our study's objectives. In response to your points, we would like to provide a more comprehensive explanation of the second goal of our study.

First, we think that our second goal is intricately linked to the first one. As we aim to shed light on potential reasons for the inconsistency in observing the Foreign Language Effect (FLe) and its relation to moral decision-making in early bilinguals, we recognize that individual differences and specific contextual factors can play a substantial role in this phenomenon. By examining the influence of psychopathic traits and variables inherent to the dilemmas, we aim to uncover the nuanced interactions that might contribute to variations in moral judgment behaviour.

Second (and fourth), we acknowledge the importance of clarifying and providing definitions for the dilemma variables. To address this, we introduce and define personal force, benefit recipient and avoidability earlier in the manuscript (page 3 lines 66-73, page 6 lines 127-129 and 132-134 of the clean copy), ensuring that readers have a clear understanding of this variables when they are later discussed in relation to Objective 2.

Third, regarding the phrasing "separately and/or jointly," we intended to investigate how psychopathic traits and dilemma variables independently impact moral judgment responses and, also, if there is an interaction between these factors. Certainly, "or" is not needed (page 8 line 201).

Fitfth, your observation regarding the introduction of difficulty, verisimilitude, and vividness is well-taken. We included these variables in our effort to obtain a complete picture about the possible factors that could influence moral decisions. From the language in which they are written, certain personality characteristics of the participants, to different subjective assessments that they could make about the dilemmas. In the case of difficulty, it has been used as measure of the emotional load and inner conflict involved in the moral choice (e.g., Carmona-Perera et al., 2013). Regarding vividness, its inclusion was based on the finding that the use of a foreign language can reduce mental imagery (Hayakawa & Keysar, 2018) and, therefore seemed interesting for the study at hand. Also, it should be noted that some of the hypothetical dilemmas used have a content that is quite far away from the reality that people experience in their daily life (war scenarios, scuba diving, rescues...). Taking into account the potential divergence between hypothetical and real moral choices (FeldmanHall et al., 2012), we decided to control how the perceived versimilitude could affect moral decisions. We now provide a more detailed explanation of why these variables were selected and how they align with our theoretical framework, enhancing the reader's comprehension of their role in our study (page 7 lines 169-176 of the clean copy).

Lastly, it is essential to clarify that Objective 2 does not aim to replicate previous findings. While it builds upon existing research in the field of psychopathic traits and moral decision-making, it extends the investigation by examining these factors within the context of the FLe and bilingualism. We recognize that understanding the interplay between psychopathic traits and dilemma variables in the presence of FLe can contribute to a more comprehensive understanding of moral judgment behavior in bilingual individuals.

We hope that this expanded explanation and revised introduction addresses your concerns and helps to better align this objective with our theoretical framework and the overall goals of the study.

*References*

Carmona-Perera, M., Martí-García, C., Pérez-García, M., & Verdejo-García, A. (2013). Valence of emotions and moral decision-making: increased pleasantness to pleasant images and decreased unpleasantness to unpleasant images are associated with utilitarian choices in healthy adults. *Frontiers in Human Neuroscience*, *7*, 626. doi:10.3389/fnhum.2013.00626

FeldmanHall, O., Mobbs, D., Evans, D., Hiscox, L., Navrady, L., & Dalgleish, T. (2012). What we say and what we do: The relationship between real and hypothetical moral choices. *Cognition*, *123*, 434-441, doi:10.1016/j.cognition.2012.02.001

Hayakawa, S. & Keysar, B. (2018). Using a foreign language reduces mental imagery. *Cognition*, *173*, 8-15. doi: 10.1016/j.cognition.2017.12.010.

- *Method*
  *Participants*
  How did you make sure that your participants were balanced bilinguals? Just by asking them?

**Response:** Participants' self-reported bilingualism was indeed a primary criterion for inclusion in the study Although language level was not objectively confirmed using other measures than a self-report, we are very confident that all participants were high proficient bilinguals due to several reasons. First, they all reported to have a high proficiency in the oral and written domains, both in the productive and the receptive areas. Second, all participants were university students. This situation obliges all participants to have a high degree of knowledge in both languages, given that the entrance exam to the University includes language level tests in both languages. Also, university classes are taught in both Catalan and Spanish, and even the same subject has professors who teach their parts either in Catalan and/or Spanish, as Catalan and Spanish are both co-official languages. Finally, using self-reports without objective measures of language level -although we concur that this is a caveat and might have its limitations- is a common practice in studies addressing the Fle (see Brower, 2019; Costa et al., 2014a; Costa et al 2014b; Keysar, Hayakawa & An, 2012; Dylman & Champoux-Larsson, 2020; Woumans, Van der Cruyssen & Duyck, 2020, among others).

*References*

Brouwer, S. (2019). The auditory foreign-language effect of moral decision making in highly proficient bilinguals. *Journal of Multilingual and Multicultural Development*, *40*(10), 865-878. doi:10.1080/01434632.2019.1585863

Costa, A., Foucart, A., Hayakawa, S., Aparici, M., Apesteguia, J., Heafner, J. et al. (2014). Your Morals Depend on Language. *PLoS ONE*. *9,* e94842. doi:10.1371/journal.pone.0094842

Costa, A., Foucart, A., Arnon, I. Aparici, M., Apesteguia. (2014). “Piensa” twice: On the foreign language effect in decision making. *Cognition*. *130*(2)*, 236-254*. doi:10.1016/j.cognition.2013.11.010

Dylman, A., & Champoux-Larsson, M. F. (2020). It’s (not) all Greek to me: Boundaries of the foreign language effect. *Cognition*, *196*. doi:10.1016/j.cognition.2019.104148

Keysar, B., Hayakawa, S. L., & An, S. G. (2012). The Foreign-Language Effect: Thinking in a Foreign Tongue Reduces Decision Biases. *Psychological Science*, *23*(6), 661–668. <https://doi.org/10.1177/0956797611432178>

Woumans, E., Van der Cruyssen, I., & Duyck, W. (2020). Crime and punishment: Morality judgment in a foreign language. *Journal of experimental psychology. General*, *149*(8), 1597–1602. doi:10.1037/xge0000736

- “Gender” would be preferable to “sex”.

**Response:** Thank you for pointing out this important distinction. We agree that "gender" is a more appropriate term than "sex" in this context. We have changed it in the manuscript (page 11 line 243 of the clean revised manuscript).

- You consider that Catalan was participants’ L1. However, is Catalan the language that the participants were mainly exposed to and communicated in? Descriptive statistics were slightly higher for L2, which makes one think that Spanish might have been the dominant language.

**Response:** Thank you for the comment. In the case of our participants, the classification of Catalan as their L1 was based on self-reporting, reflecting the language spoken within their families or communities. Moreover, except one of the participants, they were also more proficient in L1. However, a lot of bilingual people in Mallorca consider that they are equally or more proficient in L2 because their true mother tongue (Catalan spoken in Mallorca) differed from the Catalan normative language. Thus, if some of our results suggest that our participants might have been more dominant in Spanish is due to their sociolinguistic context. To provide additional information about this, in our revised manuscript we highlight in the text that, while early bilinguals can attain high proficiency in both languages, their relative proficiency in L1 and L2 can be influenced by contextual factors (page 10 lines 239-242 of the clean revised manuscript).

- 75% of the participants were females. We know that gender differences may exist when it comes to moral decision-making. How did you control for gender differences?

**Response:** To be honest, we didn't explicitly control for gender differences due to the limited sample size. We also think that, this way, we maintain clear the focus of our study.

- *Materials*
  Please define “boldness”, “meanness” and “disinhibition”.

**Response:** These constructs from the triarchic conceptualization of psychopathy are now described in the revised version of the manuscript (page 12, lines 257-267 of the clean revised manuscript). Thank you for raising up this concern.

- Please upload the dilemmas and scales on OSF. Please correct me if I am wrong, but in the link you provide only a .sav file is included, and therefore it is impossible to assess the dilemmas you have used in your study.

**Response:** Thank you for bringing this to our attention. We apologize for any inconvenience you've experienced in accessing the dilemmas and scales used in our study. The files are indeed available on the OSF repository, although perhaps we failed to make it publicly available. If the issue persists, please let us know, and we'll be happy to assist you further in accessing the files. We appreciate your understanding and patience in this matter. Anyway, we have created a new link, not anonymous, hoping that it will solve any problem: <https://osf.io/4vry5/?view_only=97832849863f465d9fe79d925a63b306>

- How many situations/dilemmas did you finally use? 16 or 22?

**Response:** Thank you for your question. The task indeed included a total of 22 dilemmas, comprising 16 moral dilemmas, 4 non-moral dilemmas, and 2 practice dilemmas (one moral and one non-moral). However, for the purpose of our analyses, only the 16 moral dilemmas were included (page 15 line 349 of the clean revised manuscript).

- *Results*
  How do you explain the fact that personal and impersonal dilemmas obtained almost the same descriptive values for valence and arousal?

**Response:** Thank you for your question. We think that this similarity in descriptive values between personal and impersonal dilemmas can be attributed to the specific selection of dilemmas used in our study. As intentionality was blocked in instrumental harm, all the dilemmas were emotionally charged and evocative for participants in both personal and impersonal scenarios. We also included a comment in the discussion addressing a potential future line of research (page 29 lines 633-636 of the clean revised manuscript).

- The values for arousal were quite similar. Why did you include arousal as a covariate? And why did some models include arousal while others both arousal and valence?

**Response:** Thank you for your question. We included arousal as a covariate, following the literature on the influence of emotional arousal on moral judgment behaviour. Although arousal levels were similar between some factors (now we refer to this fact in the Discussion –page 29 lines 632-636), we thought that we must not change the planned analysis in order to test our hypotheses.

The inclusion of valence in the last model was based on our intention to explore how different factors of the dilemmas assessed by the participants might independently or jointly affect moral judgment. We wanted to examine whether emotional arousal, valence and dominance, or their interaction with other variables, such as difficulty, verisimilitude and vividness, could provide additional insights into participants' moral decisions.

- Why did you include differences in proficiency between L1 and L2 as a covariate? Usually, L1 Catalan people are highly proficient in their L2 Spanish, which they might be using even more often than Catalan (see my previous comments).

**Response:** We included differences in proficiency between L1 and L2 as a covariate in our first model because it allowed us to account for potential variability in language proficiency within our bilingual participants. While many L1 Catalan speakers in our study were indeed highly proficient in L2 Spanish, there can still be variation in the degree of proficiency, and we wanted to control for this variability to ensure that it did not confound our results.

- We decided to explore a little bit more the data” -- What was the rationale behind this decision? Doing additional analyses without a reason seems unjustified, particularly because you did not preregister your study.

**Response:** We acknowledge that this statement is unfortunate and apologize for it. The rationale, as slightly stated some words after, was to check, in a more exploratory manner, for meaningful interactions between variables that had been arose as relevant in previous analyses in order to determine which of them were the most relevant in moral decision making. We hope that the revised statement (page 25 lines 520-523 of the clean document) is clearer.

- *Discussion*
  Could you please elaborate a bit more on the following interpretation “Moreover, contrary to Wong and Ng [18] study, our results show no arousal interaction with personal force, supporting the Dual Process Model (DPM) by Greene and colleagues”?

**Response:** We appreciate your observation because we are afraid that, in our final draft, there were some sentences deleted, leading to the confusion in our statement and it has been revised (page 27 lines 581-584). We intended to contrast our study with the one by Wong and Ng because their findings showed that as arousal increased, participants made more utilitarian judgments. It seems to be contrary to the DPM. Conversely, our results did not support this pattern. We apologize for any confusion and have addressed this mistake in the current version of the manuscript. Thank you.

- You say that considering arousal is crucial, but you also add that arousal did not modulate the effect of other predictors, nor did it predict the amount of time consumed, so taking into account arousal is crucial “when”?

**Response:** The main claim in our study is that meanness is a key factor in moral decision-making, even above other factors like arousal. However, although arousal cannot predict the response and don't modulate the effect of other predictors, it does interact with meanness. Therefore, our assertion that considering arousal is crucial pertains to situations where arousal interacts with meanness (page 28 lines 605-609). Specifically, when arousal is low, meanness predicts utilitarian responses, but as arousal levels increase, the frequency of utilitarian responses decreases (Fig2). This highlights the importance of accounting for arousal when investigating the interplay between meanness, psychopathic traits, and moral decisions.

- Most part of the discussion repeats the results and includes comparisons with previous findings, i.e. interpretations of your results are missing. For example, how do you explain the fact that your participants tended to respond more utilitarianly when they perceived more control and when the decision was perceived as more difficult? How do you explain the fact that dilemmas perceived as difficult and vivid led to slower DT? How do you interpret the interaction of boldness and dominance, or the interaction of boldness, meanness and disinhibition?, etc. As I mentioned previously, you have tried to combine and simultaneously analyse many different variables, and as a result elaborating a coherent and in-depth discussion of the main findings appears to be very challenging.

**Response:** We appreciate your thoughtful feedback and understand your concern regarding the need for a more comprehensive interpretation of our results. In response, we will provide a more detailed analysis of the main findings and their implications.

The tendency for participants to respond more utilitarianly when they perceived greater control aligns with the DPM. In our study, increased control may have led participants to engage in more deliberate processing, as they felt less inner conflict, and respond accordingly to a lesser harm decision. It is congruent with previous literature. Precisely, the perception of difficulty is related with dominance (control) assessment, but its interpretation could be a little bit tricky because the variable refers to the difficulty in making the decision. Thus, when participants engage in more extensive cognitive processing, it results in a higher likelihood of utilitarian responses. We addressed this aspect in the revised manuscript (page 29 lines 637-645 of the revised manuscript).

The finding that dilemmas perceived as difficult and vivid led to slower decision times (DT) could be explained in the same manner. As these factors are related with additional cognitive resources during the decision-making process, difficult dilemmas may require more time to be responded, and vivid dilemmas, evoking stronger emotional responses, might also slow down the decision-making process. Now we explain that in the manuscript (page 30 lines 662-665).

The interaction effects of boldness with dominance have to be with the fact that dominance refers to strong self-confidence and tolerance for unfamiliar and risky circumstances, for instance. Thus, boldness and dominance are variables measuring two related factors: the tendency to be in control of any situation (boldness) and the perceived emotional control that specific dilemma (dominance). We also take this explanation into account in the Discussion (page 31 lines 683-686).

We're not sure of what do you mean by the interaction of boldness, meanness and disinhibition. Perhaps you refer to the interaction of boldness and meanness with personal force, and disinhibition with benefit-recipient factor, when predicting the DT. The fact that all the psychopathic traits lead to faster responses to personal (and self-beneficial) dilemmas seem to be congruent with our main claim. Our results support the tendency to respond quicker to personal (and self-beneficial) dilemmas but also contribute to the literature by highlighting the need to control for psychopathic traits.

In summary, we acknowledge the need for a more thorough and coherent interpretation of our main findings. Thus, we revised the Discussion section to provide a more in-depth analysis of the results. We appreciate your feedback, which contributed to enhancing the quality of our manuscript.

- I would also like to add that what you have found in your study is similar to what has been found in previous studies with unbalanced bilinguals and realistic moral dilemmas, i.e. the lack of the moral foreign language effect. In other words, the moral language effect is not the "norm", unless unrealistic moral dilemmas (e.g. the footbridge dilemma) are used, and between this is something that you would like to elaborate on in your paper; see, for example:
  • Białek, M., Paruzel-Czachura, M., & Gawronski, B. (2019). Foreign language effects on moral dilemma judgments: An analysis using the CNI model. Journal of Experimental Social Psychology, 85, Article 103855.
  • Kyriakou, A., & Mavrou, I. (2023). ¿Eres muy emocional? I don’t think so. How does language determine our emotional responses to everyday moral dilemmas? In A. Blanco Canales & S. Martín Leralta (Eds.), Emotion and identity in second language learning (pp. 297–321). Peter Lang.
  • Muda, R., Niszczota, P., Białek, M., & Conway, P. (2018). Reading dilemmas in a foreign language reduces both deontological and utilitarian response tendencies. Journal of Experimental Psychology: Learning, Memory, and Cognition, 44(2), 321–326.

**Response:** Thank you for pointing out these additional studies. In our revised manuscript, we incorporated references to these studies and elaborate on the implications of our findings within the broader context of FLe research. Your suggestion has greatly contributed to enriching the discussion and contextualizing our results. Thank you for your valuable input.

- *Language*
  The manuscript would benefit from a proofreading (e.g. “considered themselves bilingual*”, “were neither* significant predictors”, “let us to* find”, “both a high experience of unpleasantness and a high experience of pleasantness leads* to ..”, etc.).

**Response:** Thank you for your feedback regarding language issues in the manuscript. We acknowledge the importance of proofreading, and we have carefully reviewed the entire manuscript to correct the errors you have pointed out. Your attention to detail is greatly appreciated.

- I am aware that I have raised many concerns, but I am convinced that if the authors clearly explain the contribution of their study and justify some methdodological decisions and interpretations, this paper could be a nice contribution to the literature on the moral foreign language effect.

**Response:** We really appreciate all the Reviewer#3 comments that we believe have improved the manuscript.

As a last comment to this rebuttal letter, we sincerely appreciate the time and effort you have invested in reviewing our manuscript. We have carefully addressed each of your comments and suggestions, making necessary revisions to the manuscript to ensure its accuracy and comprehensibility. We are confident that the revised version now better aligns with the standards and expectations of the journal.

Thank you again for your time and consideration.

Sincerely,

Albert Flexas

Department of Applied Pedagogy and Psychology of Education, University of the Balearic Islands (Spain). E-mail: [albert.flexas@uib.eu](mailto:albert.flexas@uib.eu).
