# Supplementary material for: Meanness trumps language: Lack of foreign language effect in early bilinguals’ moral choices
Source: PLoS One. 2023 Nov 30;18(11):e0294523. doi: 10.1371/journal.pone.0294523 (PMC10688712; doi:10.1371/journal.pone.0294523)
Supplement: Attachment — Submitted filename: Response to reviewers.docx [file pone.0294523.s002.docx]

Dear Anastassia Zabrodskaja (Academic Editor), Farzaneh Shakki, and Andres Molero-Chamizo (reviewers),

We sincerely appreciate your new comments. Below, we provide a response, outlining the minor revisions we have made.

Journal Requirements

Please review your reference list to ensure that it is complete and correct. If you have cited papers that have been retracted, please include the rationale for doing so in the manuscript text, or remove these references and replace them with relevant current references. Any changes to the reference list should be mentioned in the rebuttal letter that accompanies your revised manuscript. If you need to cite a retracted article, indicate the article’s retracted status in the References list and also include a citation and full reference for the retraction notice.

**Response:** Thank you for the reminder. We have reviewed the reference list to ensure its accuracy and we corrected a few minor mistakes (references 3, 9, 22, 24 and 41). All of them are highlighted in the manuscript with tracked changes. In response to the Reviewer #2’s comments, we included a new reference, which has also been marked as a change. In regards of your concern about retracted papers, to our knowledge, none of the papers we have cited have been retracted.

# Review Comments to the Author

Reviewer #1: Hi. Thank you for the revised files. The comments are done carefully. I am happy with the revised file.

**Response:** Thank you very much. We are glad that Reviewer#1 feels satisfied with our revised file.

Reviewer #2: The authors have responded to each of my comments, and I highly appreciate that. However, I still have a concern regarding the authors's perception of the two languages evaluated in their study. They state that Catalan (that they considered L1) is a "native language" for the participants of the study, and Spanish is an "early acquired yet distinct (not native) language". And, if I understood properly, this perception was based on self-reports, and was supposedly "confirmed" by language proficiency scores. I believe that authors should tone down a bit more these statements through the article, and do not assume that slightly higher proficiency scores are a reliable proof or evidence of the distinction between "native" and "not native" languages. This has also been criticized by reviewer 3, and I think that the authors should reflect on this limitation regarding which language is "native" or not. Obviously, Spanish is the native language in Spain.

A last minor question I have to the authors is if the 46 years old participant was also university students, as the authors state that all participants of the study were university students. If not, and considering the huge age deviation with respect to the rest of the participants, would not be more precise, statistically and echologically, only include in the analysis the young sample? If the authors keep in the analysis, for any reason, the data of the 46 years old participant, this limitation should also be discussed in the Discussion section.

**Response:** We thank Reviewer#2 for his endorsements. We understand the Reviewer’s point in terms of the differentiation between the two languages evaluated in the present study. We concur that the term ‘native language’ might not be the best way to refer to the L1 of our participants, and to establish a differentiation with Spanish, as a non-native one might not be an adequate approach. Although we inaccurately use these terms in a response to reviewer, please note that in the manuscript, we refrain from using the differentiation ‘native’ and ‘non-native’ when referring to our participants, results, and conclusions. We only use the term ‘native’ when referencing prior studies that have explicitly done so in their design, rationale or discussion (see Costa, Vives & Corey, 2017; Woumans et al., 2019).

In this vein, we revised the manuscript to ensure that we avoid to use the term ‘native’ and we only use the terms ‘L1’ and ‘L2’ to express that participants themselves (as explicitly answered by them) considered Catalan as their L1 and Spanish as their L2. Moreover, the self-reported nature of the information regarding which is the first and second language is now acknowledged as a limitation in the revised manuscript (along with the self-reported nature of proficiency ratings).

Finally, as the Reviewer correctly supposes, the 46-year-old participant was also a university student, as we state in the manuscript. Thank you for making us ensure that all of our participants were university students and double-checking this. Regarding this, we did not have an a priori strong hypothesis about age effects on the FLe so the inclusion of older adults was not perceived as a serious issue by us. At this point, we are prone to keep this participant (and others of 44, 32, 29, 28... years of age) in order to maximize sample size and guarantee that analyses focusing on our main research questions have sufficient statistical power. Despite this, we have now included a short comment about this potential limitation in the discussion section of the revised manuscript where potential age effects on moral judgment are briefly commented (McNair et al., 2019).

References:

Costa A, Vives M.L, Corey JD. On language processing shaping decision making. Current Directions in *Psychological Science*. 2017;26: 146–151. doi:<https://doi.org/10.1177/0963721416680263>

McNair, S, Okan, Y, Hadjichristidis, C, & Bruine de Bruin, W. (2019). Age differences in moral judgment: Older adults are more deontological than younger adults. *Journal of Behavioral Decision Making,* *32*, 47–60. https://doi.org/10.1002/bdm.2086

Woumans E, Van der Cruyssen I, Duyck W, Woumans E. Crime and Punishment: Morality judgment in a foreign language. Journal of Experimental Psychology: General. 2019. doi:<https://doi.org/10.1037/xge0000736>

As a last comment to this rebuttal letter, we want to thank you again for your time and consideration.

Sincerely,

Albert Flexas  (on behalf of all the authors)

Department of Applied Pedagogy and Educational Psychology, University of the Balearic Islands (Spain). E-mail: [albert.flexas@uib.eu](mailto:albert.flexas@uib.eu)
